# Energy Expenditure of Disaster Relief Operations Estimated Using a Tri-Axial Accelerometer and a Wearable Heart Rate Monitor

**DOI:** 10.3390/ijerph20095742

**Published:** 2023-05-08

**Authors:** Nao Koizumi, Hitomi Ogata, Yutaro Negishi, Hisashi Nagayama, Miki Kaneko, Ken Kiyono, Naomi Omi

**Affiliations:** 1 Institute of Health and Sport Sciences, University of Tsukuba, 1-1-1 Tennodai, Tsukuba 305-8574, Japan; 2 Graduate School of Humanities and Social Sciences, Hiroshima University, Hiroshima 739-8521, Japan; 3 Graduate School of Engineering Science, Osaka University, Osaka 565-8531, Japan

**Keywords:** rescue activities, fire fighter, energy expenditure, tri-axial accelerometer, wearable heart rate monitor

## Abstract

The management of nutrition, food, and health for disaster relief personnel is one of the crucial aspects for carrying out effective rescue activities during large-scale natural disasters, such as a big earthquake, flooding, and landslide following heavy rainfall or man-made disasters, such as widespread fire in industrial areas. Rescue workers, such as fire fighters and rescue teams who work on the disaster relief operations, have to work long, hard, and irregular hours that require energy (both intake and expenditure), with especially altered eating patterns. Reliable estimates of the energy expenditure (TEE) for such disaster relief operations have not been fully established. Here, we propose to clarify the energy expenditure for each type of large-scale disaster activity conducted by fire fighters. Thirty fire fighters (survey participants in this research) who participated in the simulation training of large-scale disaster activities wore tri-axial accelerometers and heart rate monitors during training; and, post-training, 28 fire fighters submitted complete activity record tables. An estimation formula combining tri-axial accelerometer and heart rate monitor data was used. Additionally, energy expenditure per hour (excluding resting energy expenditure: REE) (per average body weight of participants) was calculated for 10 types of large-scale disaster response activities. We propose utilization of these data as a reference value for examining the TEE of firefighting and rescue operations in future large-scale disasters.

## 1. Introduction

There have been many natural large-scale disasters, such as big earthquakes (e.g., the 3.11 Great East Japan Earthquake that almost shook the whole country), massive flooding and landslides caused by heavy rains, and man-made fire disasters (large-scale fires) in Japan. All these disasters require an immediate response by the disaster response team; usually the fire fighters are at the forefront in search and rescue operations. Fire fighters are required to promptly carry out diverse life-saving and firefighting activities as fast as possible and within 72 h of the disaster, a time which is critical to save lives under the harsh environment. To do so effectively, fire fighters energy levels are an important aspect to be considered and therein are the topic of the current research—‘energy expenditure’ of fire fighters. Although the energy expenditure of firefighting and rescue operations has been partially investigated, such as ladder climbing and lowering and firefighting activities [1,2], to the best of our knowledge, no studies are available on estimating the energy expenditure of each type of disaster relief operations during a large-scale disaster. In addition, since fire fighters play various roles in large-scale disaster relief operations, such as rescue, firefighting, emergency medical service (EMS), and logistical support activities, the equipment and intensity of activities differ greatly depending on the type of activity undertaken [3]. Such differences have made it difficult to achieve reliable energy expenditure estimation.

Previously, to demonstrate a standard value of energy expenditure for a given physical activity, Ainsworth et al. (2011) presented 821 activities [4,5,6]. Therein, the activities of fire fighters have been described by four types: (1) general; (2) rescue victim, automobile accident, using pike pole; (3) raising and climbing a ladder with full gear, simulated fire suppression; and (4) hauling hoses on the ground, carrying/hoisting equipment, breaking down walls, etc., while wearing full gear. There are studies, such as of the rescue of rescuers with ladder lifting and lowering and fire-fighting activities involving ladder lifting and lowering by expiratory gas analysis [1,2]. However, since the types of firefighting and rescue activities in large-scale disaster relief operations are not limited to single movements, such as fire extinguishing and ladder raising and lowering that are performed in normal operations, but also a wide range of activities, such as rescue activities from collapse sites and rescue activities from sediment burial sites due to landslides, it is necessary to consider more types of activities.

The gold standard for estimating energy expenditure is the Doble labeled water (DLW) method, but it is difficult to estimate the energy expenditure of a particular day by estimating the average of multiple days as the total energy expenditure per day, and it is not possible to cut out the time [7]. Exhaled gas analysis (or a douglas bag to collect expiratory gas) is another method of carrying out estimation in the laboratory, but the range of activities is restricted [7]. As alternative method that can reduce the time and can examine each (diverse) activity type, and it does not place much restriction on the activity, and the accelerometer and heart rate methods have been considered for measuring the energy expenditure using wearable devices. As a method of measuring disaster activity, corresponding to multifaceted damage in a large-scale disaster, activities that are closer to the actual (ground situation) were considered, as they are difficult to reproduce in the laboratory condition. Therefore, the effectiveness of using a wearable device that is low in invasiveness and can be adapted out in the field to actual disaster activities by fire fighters was considered. For example, a study on physiological changes during the activities of Canadian mine rescue workers used wearable devices [8], as well as in studies for a comparison of fire fighters’ activity during on-duty and off-duty [9].

In recent years, wearable devices that are less invasive, small, and easy to carry have been developed, and the accuracy of these has also been discussed in comparison with the DLW method and chambers [10,11]. In estimating energy expenditure, each method has its own limitations. Therefore, it has been suggested that combining multiple methods enables more accurate studies [12]. Studies have seen the use of accelerometers in surveys of physical activity of fire fighters. As a result of measuring the energy expenditure of firefighting activities using the accelerometer (AC) and heart rate (HR) methods through wearable devices, it was possible to confirm the activities that are not reflected in the numerical value by the AC method, but which are reflected in the HR method, and by combining the AC method and the HR method, underestimation could be generated in the estimation using the AC method alone [13]. In addition, it has been reported that fire fighters were able to estimate energy expenditure using a combination of a tri-axial accelerometer and a wearable heart rate monitor [14].

With this background, using an estimation formula combining a tri-axial accelerometer and a wearable heart rate monitor, the energy expenditure per hour for each activity type during large-scale disaster activities was estimated. The purpose of this study is to ultimately calculate it as a reference value that can be used as a standard value for each type of activity when estimating energy expenditure during future disaster activities.

## 2. Materials and Methods

### 2.1. Approach

Using a tri-axial accelerometer, a wearable heart rate monitor, and activity records, this study examined the energy expenditure of firefighting and rescue activities in large-scale disaster activities by activity type. With the approval of the Ethics Committee of University of Tsukuba, the purpose and details of the survey were explained to survey respondents, their affiliated organizations, the training management organizations, etc., and the results of the survey were conducted after obtaining their consent. (Number: T28-66, Study on the activity and nutrition of Fire service in disaster response, approved 20 August 2018).

### 2.2. Survey Target Training and Participants

Thirty fire fighters were randomly selected from among the fire fighters participating in the training to ask for cooperation in the operation to the extent that it would not interfere with the training. The fire fighters were male, selected as part of the training members of the National Fire Service Team for Disaster Response, were members who were in good health, passed the physical fitness test at the time of hiring, and had completed basic education (excluding those who have been in service for less than half a year). Each activity group consisted of five people, and measurements were made in six groups. Although one would have preferred to do this investigation in an actual disaster situation, it is not possible to predict when an actual large-scale disaster will occur. Moreover, it is very difficult to conduct such a survey during a disaster; therefore, the survey was conducted during a two-day large-scale disaster simulation drill that conducts activities largely similar to actual disasters.

### 2.3. Procedures and Instrumentation

#### 2.3.1. Study Design

The survey was conducted during a training session conducted in Kanagawa Prefecture from 30 November to 1 December 2018. Between 8:30 a.m. and 11:00 a.m., the survey respondents were each provided with a tri-axial accelerometer (Omron activity meter Active style PRO HJA-750C—23 g, 40 × 52 × 12 mm; OMRON, Kyoto, Japan) and wearable heart rate monitor (myBeat (WHS-1, myBeat, 14 g, 39 × 37 × 9 mm; Union Tool Co., Tokyo, Japan), which they carried around measuring in real-time. The average temperature was 12.8 °C (min 8.5–max 18.3 °C), and average humidity was 55.5%. After completion of the training, activity records were collected from each survey participant.

The target drill was conducted on a two-day disaster relief operation, assuming an earthquake with a maximum depth of 7.3 and a maximum depth of 6 or higher. The drill was conducted under the assumption that the earthquake caused building collapse, fire, road damage, landslides, etc., and that a large-scale fire had occurred due to damage to the complex facilities in the coastal area. A total of 2323 people participated, including 1402 from within the disaster-stricken area and 921 from outside the prefecture as an emergency fire rescue team.

The main activities were as follows: (1) rescue activities under special circumstances or special rescue activities (rescue from special situations, such as collapsed buildings and landslides); (2) rescue activities using materials, such as large heavy machinery (rescue from tunnel collapses and vehicle lockdowns, etc.); (3) large-scale fire-fighting activities; and (4) nuclear, biological, chemical (NBC) disaster response and rescue activities (rescue from scientific disasters).

#### 2.3.2. Placement of Tri-Axial Accelerometer and Wearable Heartrate Monitor

The tri-axial accelerometer was taped to the bottom of the inside of the left breast pocket (10 cm above the waist) in a transparent plastic bag to prevent flood failure, as in previous studies [15]. The wearable heart rate monitorwas fastened with Velcro to the center of the inner chest where conductive fibers are woven (manufactured by Kurabo Industries Ltd., Osaka, Japan) [14].

#### 2.3.3. Accelerometer

A tri-axial accelerometer (Active Style Pro HJA-750C, 23 g, 40 × 52 × 12 mm; Omron Healthcare Corporation) was used as in a previous study [15], which actually measured the energy expenditure of fire and rescue operations using wearing wearable devices. The accelerometer was programmed to save the transformed metabolic equivalents (METs) using Omron’s algorithm once every 10 s [16,17], and, as in previous studies, the average of one minute was calculated [15]. Basal metabolism was calculated automatically upon entering age, sex, and weight [16,17].

#### 2.3.4. Wearable Heartrate Monitor

The myBeat (WHS-1, myBeat, 14, 39 × 37 × 9 mm, 14g; Union Tool Co., Tokyo, Japan) used in a previous study [14], in which the heart rate of fire and rescue operations was actually measured by wearing a wearable device on a subject. A heart rate monitor was attached to the center of the inner chest where conductive fibers manufactured by Claveaugh were woven and measured. As in previous studies, we adopted a median value that allowed heart rate to be measured by 50% or more per minute, excluding measurement errors [14].

#### 2.3.5. Estimation Formula

##### Calculation of Energy Expenditure during Disaster Relief Operations

Data obtained from the tri-axial accelerometer and heart rate monitor were synthesized. Using the following estimation formula [14],

(1) $${\mathrm{METs}}_{\mathrm{act}}=\frac{{\mathrm{MET}}_{\max}-{\mathrm{MET}}_{\min}}{{\mathrm{HR}}_{\max}-{\mathrm{HR}}_{\min}}\left({\mathrm{HR}}_{\mathrm{act}}-{\mathrm{HR}}_{\min}\right)+{\mathrm{METS}}_{\min}$$

the MET values and energy expenditure of each activity were estimated. In this equation, two representative reference points $\left({\mathrm{HR}}_{\min},{\mathrm{MET}}_{\min}\right)$, $\left({\mathrm{HR}}_{\max},{\mathrm{MET}}_{\max}\right)$, respectively, corresponding to minimum and maximum activity of the individual were used to create the individually optimized estimation formula of the MET value, ${\mathrm{METs}}_{\mathrm{act}}$, based on the measured HR, ${\mathrm{HR}}_{\mathrm{act}}$ [14]. The minimum point was a point that corresponded to the 10th percentile heart rate during the nap to 0.95 METs of sleep in the METs table [6], and the maximum point was the point where the activity with the highest 90th percentile heart rate in disaster relief operations was associated with the MET value, corresponding to this activity in the MET table [6]. Using individually created trend lines, combined MET values were calculated every minute from the heart rate during disaster relief operations and converted to the energy expenditure [14]. The conversion from MET values to energy expenditure was performed using Omron’s conversion formula [16,17].

Calculation of Reference Data for the Hourly Energy Expenditure during Individual Disaster Relief Operations

The same activity was identified from the activity log. The average value per hour was calculated from the energy consumption during the activity obtained by the method in Calculation of Energy Expenditure during Disaster Relief Operations and used as the reference data for each activity.

#### 2.3.6. Activity Log

Each group was asked to distribute a sheet of record paper and explain what activities they had performed and when during the disaster simulation drill. Activities were filled out every 30 min, and the categories of movement, waiting, nap, rest, meal, firefighting, emergency activities, rescue activities, and logistical support activities were selected, and details of the activities could be described separately. This recording paper was used to classify the types of activities during activity hours (Supplemental Data Table S1).

#### 2.3.7. Analysis

All data used the mean and standard deviation. The data for the tri-axial accelerometer is downloaded in a dedicated application, and the data for the wearable heart rate monitor are available in R, version 3.6.0 (R Foundation for Statistical Computing, Vienna, Austria. http://www.R-project.org/, accessed on 8 February 2023).

## 3. Results

### 3.1. Survey Respondents

We surveyed 30 subjects (fire fighters). However, as two subjects had defects in the data (during data collection period), which was due to technical issues/equipment failure, they were excluded from the results; the data of 28 subjects (Table 1) were analyzed. The survey respondents were divided into six groups of five people from each group, and they were active from 11 a.m. to the next 11 a.m. The simulation training started at 8:30 a.m., and almost all teams (research subjects therein) arrived from their base by vehicles at the training site around 11 a.m. and started the activity.

### 3.2. Details of the Activities each Activity Group

**Figure 1:**
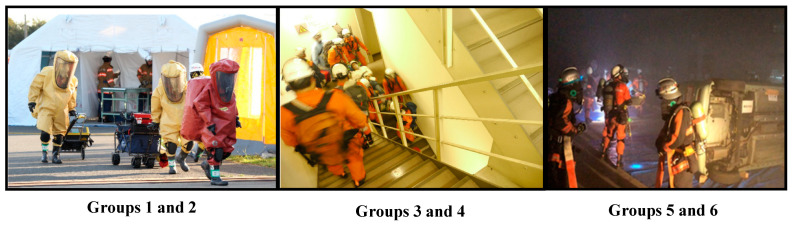
Photos for each firefighting activity illustrate the difference in scenarios and equipment’s used. Depending on the equipment, there are differences in weight, ease of breathing, ease of movement, etc. Activities of Groups 1 and 2 corresponded to a gas leak accident at the complex. They wore protective clothing sealed over their usual equipment to avoid inhaling toxic gases and are carrying oxygen cylinders. Activities of Groups 3 and 4 involved climbing staircases where they had to go up and down the floors of a high-rise building as rescue personnel. They climbed the stairs to the upper floors with their usual equipment, and after the rescue operation, they went down the stairs while transporting people in need of rescue on stretchers and on their backs. Activities of Groups 5 and 6 show engagement in landslide disaster activities and tunnel collapse accident relief activities. In the tunnel collapse accident rescue operation, the activity was carried out with an oxygen cylinder on their back so as not to inhale the toxic gases and dust in the tunnel.

The METs and total energy expenditure for each activity type by activity group are shown (Table 2). METs for each type of activity ranged from 4.3 METs to 7.0 METs. The main activities of Groups 1 and 2 were gas leak rescue (Figure 1). Due to the impact of the earthquake in the complex, buildings collapsed, facilities and equipment were damaged, and fires associated with this occurred. It was assumed that toxic gas leaks occurred on the third basement floor of the ventilation facility, and that there were several delays in escape. The survey respondents wore a protective suit that prevents the inhalation of toxic gases on top of their normal equipment (fire protection coat), and because they carried oxygen cylinders on their backs, they were able to operate under a load that was higher than normal equipment. Groups 3 and 4 were mainly responsible for the rescue from high-rise buildings (Figure 1). As expected, several employees who were conducting evacuation guidance were injured when the ceiling collapsed when an earthquake occurred, while several tourists were touring the city on the observation floor of the 69^th^ floor of a high-rise building. The elevator was disabled, and access to the upper floors was only by emergency stairs. Eight of the rescuers were able to walk, but five of them, including two seriously injured, were unable to walk, so the respondent descended the stairs from the upper floors with the load of the rescued person by stretcher transport or backpack rescue. It is to be noted that, in the above, there was one stairwell up and down to the 69th floor observation deck, and it was comprised of only one rescue training up and down the staircase. Groups 5 and 6 were mainly responsible for landslide disasters and tunnel collapse accidents (Figure 1). The landslide occurred due to an earthquake, and one passenger car was half buried in the mudslide, and one subsequent passenger car collided. It was assumed that there were injured people inside, but that the door was out of shape by the impact of the collision and could not be opened. Some large heavy machinery was used to remove soil and sand and to open and close doors. In the case of the tunnel collapse accident, when entering the tunnel, they wore oxygen cylinders and acted with load. The lights were off in the tunnel, and they operated with the light of the headlights in the midst of poor visibility.

For each group to which the participants belonged, the contents of the activities were read from the activity record sheet and categorized. The average METs value (±SD) and activity time for 1 min for each activity type are shown. Activities of Groups 1 and 2 corresponded to a gas leak accident at the complex. Activities of Groups 3 and 4 involved climbing staircases where they had to go up and down the floors of a high-rise building as rescue personnel. Fire fighters climbed the stairs to the upper floors with the equipment they normally use, and after the rescue operation, they went down the stairs while transporting people in need of rescue on stretchers and on their backs. Group 3 joined the general firefighting after skyscraper training. Activities of Groups 5 and 6 show engagement in landslide disaster activities and tunnel collapse accident relief activities. Group 5 joined the rescuer transport after the landslide disaster activities.

### 3.3. Energy Expenditure by Type of Activity

Based on the results obtained for the details of activities in each activity group, equivalent activities were classified by activity type as a reference data of energy expenditure per hour for 10 types of large-scale disaster relief operations (Table 3). Rescue operations from small areas consumed the least energy, estimated at 249 kcal per hour. The activity was part of a rescue operation from the collapsed building. Firefighting activity consumed 291 kcal per hour. Gas-related accidents consumed around 327 kcal per hour. The activity was heavy due to the layering of protective clothing on top of normal activity clothes and equipment, and the load had been increased because it blocked the outside air. The tunnel collapse accident consumed an estimated 355 kcal per hour. This activity is considered to be of high risk due to toxic gas accumulating in the tunnel, so fire fighters have to wear a respirator, and breathing is restricted. It also includes high-impact activities, such as the removal of debris from tunnel collapses. The use of large heavy machinery was also confirmed in these activities. Rescue operations from high altitudes showed the highest energy expenditure, estimated at 419 kcal per hour. When going up the stairs, fire fighters must run with full equipment to the site as soon as possible where the victim is located. Additionally, while coming down from a higher place, an additional load is added by the presence of a victim either on their back or on the stretcher.

## 4. Discussion

Here, we demonstrated the practicality and effectiveness of a wearable device-based approach for the TEE estimation in large-scale disaster activities. The standard value of energy expenditure for each type of activity was determined using an estimation formula combining a tri-axial accelerometer and a wearable heart rate monitor. The activities of the fire fighters in large disaster are a complex combination of various activities. To estimate the energy expenditure of fire fighters’ activities, it is necessary to grasp the entire series of flows connected to each activity. The physical activity intensity of firefighting activity has been reported for each part (e.g., ladder climbing, dragging hoses on the ground) [2]. To know the energy expenditure per hour, it is possible to estimate the total energy expenditure of activities in a large-scale disaster. In this study, in a long-term drill in which multiple disaster activities are performed, the energy expenditure per hour of each activity was investigated in actual activities. This result could help our understanding for the energy expenditure of fire fighters engaged in complex disaster activities. The lowest energy expenditure activity was 249 kcal per hour (for rescue from a small area), followed by 263 kcal per hour (for rescue from a vehicle). In these activities, large heavy machinery is used to secure conductors, destroy the entrance, unlock it, etc. [19], so it is thought that energy expenditure has become low. In addition, it is conceivable that the range of activities, such as narrow areas and vehicles, was limited. The standard activity suit weighed 25 kg [15], and it is thought that the activity was relatively low in load with relatively little addition of equipment, such as oxygen cylinders outdoors. From these facts, it is thought that energy expenditure has also become relatively low.

Additionally, the highest energy expenditure activity is a rescue operation from a high place with stair lifts and descents, which is 419 kcal per hour. The next highest was tunnel collapse rescue. In the case of a tunnel collapse accident, when entering the tunnel, an air respirator is attached, so the load is considered accordingly. In both cases, the load, activity, and range of action at the time of equipment and rescue of people in need of rescue were vast, so physical activity is thought to have increased, so it is thought that energy expenditure also increased. It has been shown that the load of equipment, such as an air respirator, imposes a biological load on fire fighters [20]. Since there is a large difference in energy expenditure for each type of activity in large-scale disaster relief operations, it is thought that it will be possible to more easily estimate the total daily energy expenditure in combination with the expected activity implementation time by showing the energy expenditure of each activity as a standard value.

The total energy expenditure of fire fighters during normal duty was 3626 kcal per day in a survey of fire fighters [21], wild and firefighter. It has been reported as 4878 kcal per day in a survey [22] and 4009 kcal per day in urban fire fighters [23]. AC Law reported 4716 kcal per day for wild fire fighters [24] and 2531 kcal per day for urban fire fighters [23]. In this study, the total energy expenditure was estimated to mean 4838 (±508) kcal. In previous studies, the DLW method, which is considered as the gold standard, required a long-term investigation; therefore, it was not possible to estimate energy consumption during the activities alone. Additionally, there are no studies investigating large-scale disasters with prolonged periods of time and multiple activities using the DLW method [21,22,23]. In the case of the AC method, it is possible to measure only the activity time. The TEE of firefighters during a large-scale disaster under the AC Act was reported to be 3619 ± 499 kcal, but an underestimation was also reported [15]. In this study, the AC and HR methods were combined to estimate energy consumption only during the active hours. As a result, authors believe they were able to evaluate the part that was considered to have been underestimated by the AC method. However, by the addition of the HR method an effect on changes in heart rate related to tension and temperature changes may be seen. This aspect needs to be considered in future studies.

When analyzing activity details, such as MET values and activity time for each activity type by activity group, group 3.4 has the highest energy expenditure, and stair climbing and descending has the least activity time. TEE was found to be the lowest. This is thought to be because the squadrons that performed energy-consuming activities because of the training are adjusting their activity time and subsequent activities. In the training subject to this survey, the elevation to the upper floors only occurred once, but, in the actual disaster, it is possible that it may occur multiple times, and the activity time may be longer, so the TEE is also considered to be higher. It has been shown that the longer the time spent wearing firefighting equipment, the greater the heat stress and the greater the load on the organism than the activity in a jersey [25].

As an example, we calculated the TEE of an actual activity in a real disaster situation. During this disaster, a landslide occurred due to heavy rains, and people went missing on 3 July 2021 at Atami city in Shizuoka prefecture, Japan. This is a record of the rescue activities of second day after the disaster occurred (4 July 2021). In this rescue activity, firefighters carried out excavation activities to search for the missing people. Additionally, the TEE was estimated using data presented in Table 3 (excavation activity) and the other activities (for example, eating, moving, meeting, etc.), referring to the METs table [6]. We estimated a TEE of around 5436 kcal on 4 July 2021 (the second day of the disaster). By utilizing the results of this study and estimating the energy consumption of even more activities, it is expected that it will lead to an understanding of the energy expenditure of firefighters. Until now, the energy expenditure for each large-scale disaster activity has not been clarified. From the present research, it is expected to be able to estimate the energy expenditure for each type of disaster based on the activity records so far. In addition, according to the estimated energy expenditure, authors propose utilizing it for managing the amount of energy in the food to be replenished and for activity management, such as rotation of the crew members.

The activity training investigated in this study comprises the widest range of training currently measurable and is the longest running exercise currently conducted in Japan. This is also the subject of a survey, which includes a wide variety of activities. However, it is to be noted that additional investigation is necessary for activities that were not carried out in this survey target training (e.g., mountain rescue and water rescue). This study shows the importance of measurement in actual disaster activities, but it was difficult because they were not allowed to wear other equipment, such as Douglas bags, during the activity training. In order to confirm this result, it is necessary to do additional experiments in future studies using a Douglas bag and other methods. In this research, we focused on actually measuring the activities during disaster-relief operations, which are complex activities. We selected the longest running activity and targeted the maximum number of people who could be measured wearing accelerometers and heart rate monitors. However, this number of the participants is limited, and we believe that further research is needed. In addition, wearable sensors wearable sensors are less likely to interfere with training and work, and the data are easy to measure and record, so the number of examples can be increased in practical situations of fire-fighting and trainings. We did not consider the role of fatigue in this study. Since the time required varies greatly depending on the activity, it might be possible that fatigue has an effect. Moreover, the relationship between time and estimated energy expenditure per hour is not clear. Additionally, it is not known whether energy expenditure varies due to fatigue. This aspect also needs to be checked in future research. In addition, since disaster response activities were carried out throughout the day and night, it is necessary to consider the circadian rhythm in addition to the changes in heart rate and metabolism in the future.

Survey respondents were randomly selected from the members active in the target training, but since women were not included in the participating personnel, only men were included. This may be due to the fact that there are few female members in the Emergency Fire Rescue Team currently operating in Japan, but women are gradually being registered as members, and it is conceivable that women will also be dispatched to disaster activities. For these reasons, it is necessary to conduct surveys targeting women in the future. In addition, it is possible that the activity time is adjusted because the activity content is controlled because it is an activity drill, and the activity time is adjusted due to the fact that the squad that carried out high-intensity activities longer than the actual disaster. In addition, since activity records are self-reported, there may be differences from the actual situation. The results of this study can be applied to estimate the energy expenditure in not only for the firefighters, but also for individuals (e.g., Japan Self Defense Force, Police) involved in different types of disaster relief operations.

## 5. Conclusions

Our study has investigated the TEE of fire fighters during large-scale disaster relief operations, where a reference value of energy expenditure for each type of activity in large-scale disaster relief operations was established using an estimation formula combining a tri-axial accelerometer method and a wearable heart rate method. As a result, this study clarifies values that are considered to correspond to the standard values of hourly energy expenditure for 10 types of disaster relief operations. The authors also note that, due to the limited number of samples in the present study, further research will be needed to generalize these results.

## Figures and Tables

**Table 1:** Characteristics of the survey respondents.

Number of Respondents	Age (Years)	Height (cm)	Body Weight (kg)	BMI *^1^	Basal Metabolic Rate (kcal)
Overall (n = 28 *^2^) MEAN ± SD	34.0 ± 7.4	173.4 ± 5.4	68.7 ± 5.6	22.9 ± 1.5	1544 ± 89

*^1^ BMI: body mass index = mass (kg)/height^2^ (m). *^2^ 28 subjects’ data were analyzed, and except for two individuals, who were excluded due to equipment failure, they had a mean value of age, height, weight, BMI, and basal metabolic rate (±SD; standard deviation), which are shown. Basal metabolism was calculated automatically upon entering age, sex, and weight into an Omron application [16,17].

**Table 2:** The average METs and activity time for each type of large-scale disaster relief operations activity.

Number	Activity	METs	Activity Time (min)
Group 1 (N = 5)	Rescue activity training in a gas leak accident	5.3(±1.4)	360
	Search activity	4.8 (±1.0)	30
	Rescue training in a collapsed building	4.3 (±1.0)	150
	Subtotal		540
Group 2 (N = 4)	Rescue activity training in a gas leak accident	6.2(±0.3)	210
	Rescue training in a collapsed building	4.9 (±0.7)	150
	Subtotal		360
Group 3 (N = 5)	Rescue activity training in a skyscraper	7.0 (±1.1)	120
	General rescue activity	6.0 (±0.4)	60
	General firefighting	5.1 (±0.6)	120
	Subtotal		300
Group 4 (N = 5)	Rescue activity training in a skyscraper	6.9 (±1.0)	150
	General rescue activity	6.0(±0.9)	60
	Search activity	5.0(±1.8)	30
	Subtotal		240
Group 5 (N = 5)	Excavation rescue search activity	5.5 (±1.8)	90
	Rescue training from vehicles	4.7(±1.5)	120
	Rescue training in a collapsed building	5.0 (±4.4)	60
	Rescuer transport	4.8 (±1.4)	60
	Subtotal		330
Group 6 (N = 4)	Excavation rescue search activity	6.0 (±1.1)	210
	Tunnel accident rescue activity	5.6 (±1.4)	240
	Subtotal		450

**Table 3:** Data corresponding to the average METs per minute for each activity type, including the reference data of energy expenditure per hour.

Activity Type	N	Average AC/HR METs	Min.	Max.	Reference Data of Energy Expenditure per Hour (kcal) (Adult Males Average of 71 kg *^1^ Excluding Resting Metabolism *^2^)
Rescue from a Small Place	14	4.5 (±1.2)	3.1	7.4	249
Rescue from the Vehicle	5	4.7 (±1.5)	3.6	7.3	263
Rescue Personnel Transportation	5	4.8 (±1.4)	3.1	6.9	270
Search Activities	10	4.9 (±1.3)	2.2	6.9	277
Fire Fighting Activities	5	5.1 (±0.6)	4.1	5.7	291
Gas-related Accidents	14	5.6 (±1.3)	3.1	8.1	327
Excavation Activities	9	5.7 (±1.4)	3.9	7.6	334
Rescue Operations	10	6.0 (±0.7)	5.0	6.9	355
Tunnel Collapse Accident	4	6.0 (±1.1)	4.7	7.2	355
Rescue from a High Ground (e.g., up and down the stairs)	20	6.9 (±1.0)	4.7	8.0	419

Disaster activities were classified into 10 types of activities, and reference data of the number of survey respondents, average per minute (±SD), minimum data, maximum data, and energy expenditure per hour (kcal) calculated by estimation formula were shown. The reference data were obtained by multiplying the MET data calculated from the estimation formula combining AC and HR by 71 kg, which is the average body weight of a Japanese (general) adult man, excluding resting metabolism. *^1^ According to the 2017 National Nutrition and Health Survey by the Ministry of Health, Labour and Welfare Japan, this is the average weight of men aged 30–39 years [18]. *^2^ Reference data of energy expenditure per hour (kcal) = AC/HRMETs − 1METs × 71 (kg) × 1 (hour) (Ainsworth et al., 2011).

## Data Availability

Not applicable.

## Supplementary Materials

The following supporting information can be downloaded at: https://www.mdpi.com/article/10.3390/ijerph20095742/s1, Table S1: Activity Log.
