# Supplementary material for: Energy Expenditure of Disaster Relief Operations Estimated Using a Tri-Axial Accelerometer and a Wearable Heart Rate Monitor
Source: Int J Environ Res Public Health. 2023 May 8;20(9):5742. doi: 10.3390/ijerph20095742 (PMC10178310; doi:10.3390/ijerph20095742)
Supplement: Supplementary file 1 [file ijerph-20-05742-s001.zip › ijerph-2240503-supplementary.pdf]

International Journal of
Environmental Research and Public Health

MDPI

Supplemental file Table S1. Activity Log

Activities	(circle all that apply)									Description of Activities' detail
example	moving	standby	nap	rest	meal	firefighting	emergency medical activities	rescue	logistical support	(Removing debris, carrying victims, shoveling Excavating with shovels, etc.)
8:00	moving	standby	nap	rest	meal	firefighting	emergency medical activities	rescue	logistical support	( )
8:30	moving	standby	nap	rest	meal	firefighting	emergency medical activities	rescue	logistical support	( )
9:00	moving	standby	nap	rest	meal	firefighting	emergency medical activities	rescue	logistical support	( )
9:30	moving	standby	nap	rest	meal	firefighting	emergency medical activities	rescue	logistical support	( )
10:00	moving	standby	nap	rest	meal	firefighting	emergency medical activities	rescue	logistical support	( )
10:30	moving	standby	nap	rest	meal	firefighting	emergency medical activities	rescue	logistical support	( )
11:00	moving	standby	nap	rest	meal	firefighting	emergency medical activities	rescue	logistical support	( )
11:30	moving	standby	nap	rest	meal	firefighting	emergency medical activities	rescue	logistical support	( )
12:00	moving	standby	nap	rest	meal	firefighting	emergency medical activities	rescue	logistical support	( )
12:30	moving	standby	nap	rest	meal	firefighting	emergency medical activities	rescue	logistical support	( )
13:00	moving	standby	nap	rest	meal	firefighting	emergency medical activities	rescue	logistical support	( )
13:30	moving	standby	nap	rest	meal	firefighting	emergency medical activities	rescue	logistical support	( )
14:00	moving	standby	nap	rest	meal	firefighting	emergency medical activities	rescue	logistical support	( )
14:30	moving	standby	nap	rest	meal	firefighting	emergency medical activities	rescue	logistical support	( )
15:00	moving	standby	nap	rest	meal	firefighting	emergency medical activities	rescue	logistical support	( )
15:30	moving	standby	nap	rest	meal	firefighting	emergency medical activities	rescue	logistical support	( )
16:00	moving	standby	nap	rest	meal	firefighting	emergency medical activities	rescue	logistical support	( )
16:30	moving	standby	nap	rest	meal	firefighting	emergency medical activities	rescue	logistical support	( )
17:00	moving	standby	nap	rest	meal	firefighting	emergency medical activities	rescue	logistical support	( )
17:30	moving	standby	nap	rest	meal	firefighting	emergency medical activities	rescue	logistical support	( )
18:00	moving	standby	nap	rest	meal	firefighting	emergency medical activities	rescue	logistical support	( )

18:30	moving	standby	nap	rest	meal	firefighting	emergency medical activities	rescue	logistical support	( )
19:00	moving	standby	nap	rest	meal	firefighting	emergency medical activities	rescue	logistical support	( )
19:30	moving	standby	nap	rest	meal	firefighting	emergency medical activities	rescue	logistical support	( )
20:00	moving	standby	nap	rest	meal	firefighting	emergency medical activities	rescue	logistical support	( )
20:30	moving	standby	nap	rest	meal	firefighting	emergency medical activities	rescue	logistical support	( )
21:00	moving	standby	nap	rest	meal	firefighting	emergency medical activities	rescue	logistical support	( )
21:30	moving	standby	nap	rest	meal	firefighting	emergency medical activities	rescue	logistical support	( )
22:00	moving	standby	nap	rest	meal	firefighting	emergency medical activities	rescue	logistical support	( )
22:30	moving	standby	nap	rest	meal	firefighting	emergency medical activities	rescue	logistical support	( )
23:00	moving	standby	nap	rest	meal	firefighting	emergency medical activities	rescue	logistical support	( )
23:30	moving	standby	nap	rest	meal	firefighting	emergency medical activities	rescue	logistical support	( )
0:00	moving	standby	nap	rest	meal	firefighting	emergency medical activities	rescue	logistical support	( )
0:30	moving	standby	nap	rest	meal	firefighting	emergency medical activities	rescue	logistical support	( )
1:00	moving	standby	nap	rest	meal	firefighting	emergency medical activities	rescue	logistical support	( )
1:30	moving	standby	nap	rest	meal	firefighting	emergency medical activities	rescue	logistical support	( )
2:00	moving	standby	nap	rest	meal	firefighting	emergency medical activities	rescue	logistical support	( )
2:30	moving	standby	nap	rest	meal	firefighting	emergency medical activities	rescue	logistical support	( )
3:00	moving	standby	nap	rest	meal	firefighting	emergency medical activities	rescue	logistical support	( )
3:30	moving	standby	nap	rest	meal	firefighting	emergency medical activities	rescue	logistical support	( )
4:00	moving	standby	nap	rest	meal	firefighting	emergency medical activities	rescue	logistical support	( )
4:30	moving	standby	nap	rest	meal	firefighting	emergency medical activities	rescue	logistical support	( )
5:00	moving	standby	nap	rest	meal	firefighting	emergency medical activities	rescue	logistical support	( )
5:30	moving	standby	nap	rest	meal	firefighting	emergency medical activities	rescue	logistical support	( )
6:00	moving	standby	nap	rest	meal	firefighting	emergency medical activities	rescue	logistical support	( )

6:30	moving	standby	nap	rest	meal	firefighting	emergency medical activities	rescue	logistical support	( )
7:00	moving	standby	nap	rest	meal	firefighting	emergency medical activities	rescue	logistical support	( )
7:30	moving	standby	nap	rest	meal	firefighting	emergency medical activities	rescue	logistical support	( )
8:00	moving	standby	nap	rest	meal	firefighting	emergency medical activities	rescue	logistical support	( )
8:30	moving	standby	nap	rest	meal	firefighting	emergency medical activities	rescue	logistical support	( )
9:00	moving	standby	nap	rest	meal	firefighting	emergency medical activities	rescue	logistical support	( )
9:30	moving	standby	nap	rest	meal	firefighting	emergency medical activities	rescue	logistical support	( )
10:00	moving	standby	nap	rest	meal	firefighting	emergency medical activities	rescue	logistical support	( )
10:30	moving	standby	nap	rest	meal	firefighting	emergency medical activities	rescue	logistical support	( )
11:00	moving	standby	nap	rest	meal	firefighting	emergency medical activities	rescue	logistical support	( )
11:30	moving	standby	nap	rest	meal	firefighting	emergency medical activities	rescue	logistical support	( )
12:00	moving	standby	nap	rest	meal	firefighting	emergency medical activities	rescue	logistical support	( )
12:30	moving	standby	nap	rest	meal	firefighting	emergency medical activities	rescue	logistical support	( )
13:00	moving	standby	nap	rest	meal	firefighting	emergency medical activities	rescue	logistical support	( )
13:30	moving	standby	nap	rest	meal	firefighting	emergency medical activities	rescue	logistical support	( )
14:00	moving	standby	nap	rest	meal	firefighting	emergency medical activities	rescue	logistical support	( )
14:30	moving	standby	nap	rest	meal	firefighting	emergency medical activities	rescue	logistical support	( )
15:00	moving	standby	nap	rest	meal	firefighting	emergency medical activities	rescue	logistical support	( )
15:30	moving	standby	nap	rest	meal	firefighting	emergency medical activities	rescue	logistical support	( )
16:00	moving	standby	nap	rest	meal	firefighting	emergency medical activities	rescue	logistical support	( )
16:30	moving	standby	nap	rest	meal	firefighting	emergency medical activities	rescue	logistical support	( )
17:00	moving	standby	nap	rest	meal	firefighting	emergency medical activities	rescue	logistical support	( )
